# Count data, rates, rate differences, and rate ratios in meta‐analysis: A tutorial

**DOI:** 10.1002/cesm.70022

**Published:** 2025-02-28

**Authors:** Christopher James Rose, Milena Geist, Matteo Bruschettini

**Affiliations:** ^1^ Center for Epidemic Interventions Research Norwegian Institute of Public Health Oslo Norway; ^2^ Cluster for Reviews and Health Technology Assessments Norwegian Institute of Public Health Oslo Norway; ^3^ Institute for Medical Information Processing, Biometry, and Epidemiology Ludwig Maximilian University of Munich Munich Germany; ^4^ Pettenkofer School of Public Health Munich Germany; ^5^ Paediatrics, Department of Clinical Sciences Lund Skåne University Hospital Lund University Lund Sweden; ^6^ Cochrane Sweden, Department of Research, Development, Education and Innovation Skåne University Hospital Lund University Lund Sweden

## Abstract

This tutorial focuses on trials that assess outcomes by counting events that can occur zero, one, or more than one time in each participant. Trials and meta‐analyses can estimate treatment effects for count outcomes using rate differences or rate ratios. We explain why it may be appropriate to meta‐analyze count data to estimate rate ratios rather than odds ratios, risk ratios, or risk differences. We explain what count data are, how trials may estimate treatment effects, how to interpret such estimates, and how to extract data from trials that use count outcomes for meta‐analysis. Finally, we discuss some common misunderstandings and subtleties. Supplementary materials include an Excel file for performing calculations, mathematical background, and additional advice.

## Graphical abstract

This tutorial focuses on trials that assess outcomes by counting events that can occur zero, one, or more than one time in each participant. Trials and meta‐analyses can estimate treatment effects for count outcomes using rate differences or rate ratios. We explain why it may be appropriate to meta‐analyze count data to estimate rate ratios rather than odds ratios, risk ratios, or risk differences. We explain what count data are, how trials may estimate treatment effects, how to interpret such estimates, and how to extract data from trials that use count outcomes for meta‐analysis. Finally, we discuss some common misunderstandings and subtleties. Supplementary materials include an Excel file for performing calculations, mathematical background, and additional advice. Count data and rates micro learning module

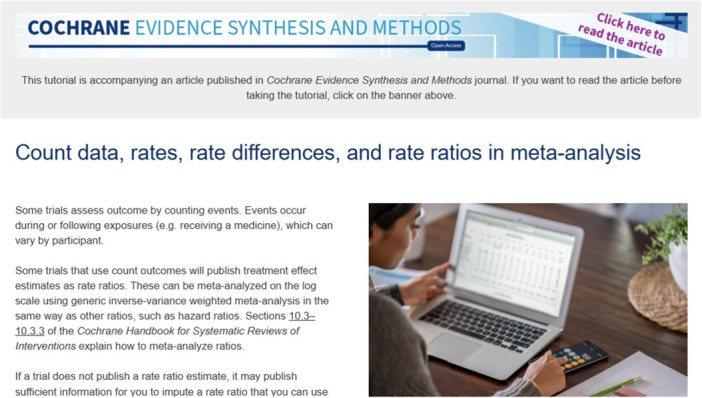

## 1 INTRODUCTION

Many trials assess outcome using dichotomous (binary) outcomes (e.g., having zero *vs.* more than zero heart attacks). Dichotomous outcomes mask the intensity of outcomes that can occur multiple times. Count outcomes capture intensity information and can be statistically more powerful than dichotomous outcomes, allowing trials to reduce sample sizes. This can have ethical and economic advantages. Reviewers need to understand counts and how to meta‐analyze trials that use them, particularly for adverse events (AEs). We use the Rhein et al. trial on withdrawal of caffeine from preterm infants with bronchopulmonary dysplasia in our examples [1].

## 2 WHAT IS A COUNT OUTCOME?

Counts are widely understood: even children who cannot yet read or write can usually tell you how many siblings they have. Counts are nonnegative integers (0, 1, 2, …): a house cannot have minus three rooms, and one cannot have one‐third of a brother.

**Figure 1:**
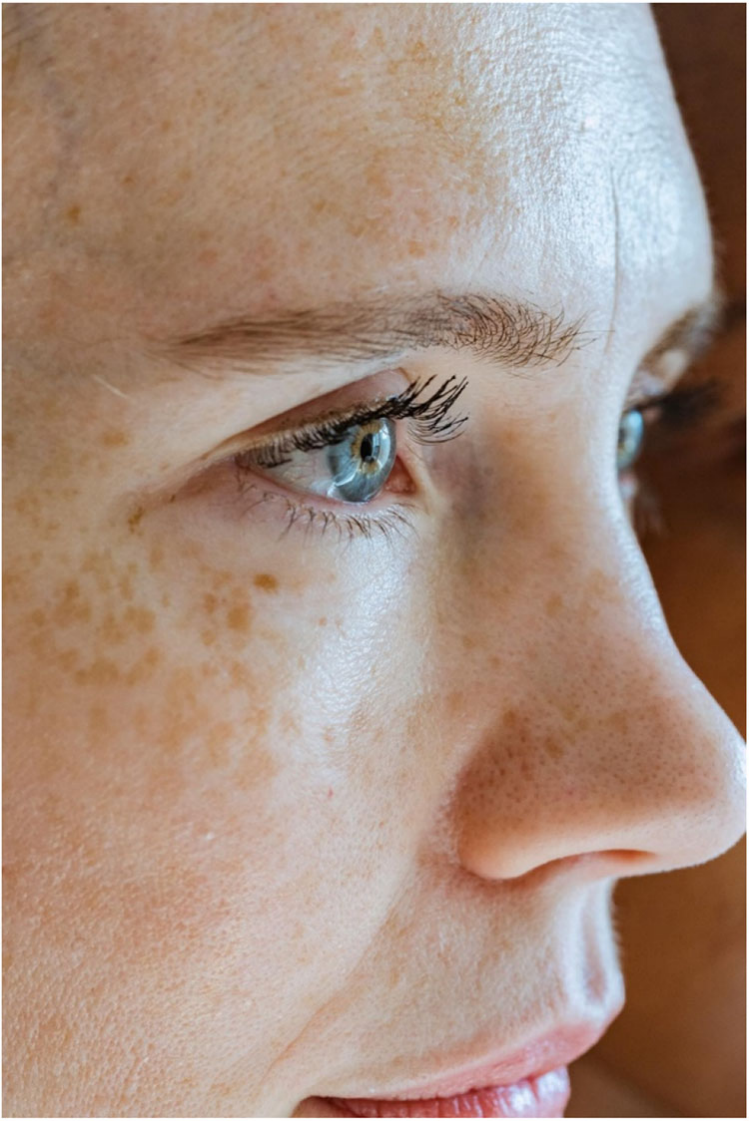
*Example of a count outcome. Freckles develop predominantly in people with Northern European genetics as a result of UV‑B exposure (e.g., sunlight). The number of freckles per 10 cm² of skin could be used as an outcome in a trial comparing sunscreen use to no sunscreen. What are the advantages and disadvantages of using this outcome, and in including such trials in a meta‐analysis? Our answer is provided in the appendix. (Photo credit:* Angela Roma).

It is essential to understand what *events* are counted, who or what they are counted *on*, and what they are counted *over*. Counts typically reflect beneficial or harmful events; *on* trial participants; *over* time. Alternatively, events may be counted *on* things (e.g., hospitals), or *over* space (e.g., square centimeters of skin; Figure 1). Rhein counted intermittent hypoxia episodes (IHEs) *on* preterm infants, *over* time.

The time or space over which events are counted is called *exposure*. Exposure can vary between participants (e.g., some participants may die earlier, so are exposed to event risk for less time). The sum of a trial arm's exposures is called *total exposure*. Count outcomes cannot be analyzed unless exposures or total exposures are measured or imputed. The combination of what events are counted *on* and *over* is called the *unit of exposure*. Rhein's unit of exposure is infant‐hour. Different trials on the same topic may use different units of exposure. This might reflect clinically important methodological heterogeneity (Cochrane Handbook [2]. §10.10) or unimportant preferences.

## 3 WHAT IS A RATE?

A *rate* (or sometimes *intensity*) is the average count per unit of exposure. Rhein's control was caffeine discontinuation (usual care), which had a rate of 8.4 IHEs per infant‐hour. While counts are nonnegative integers, rates are nonnegative real numbers. Unlike risks, rates can be greater than 1. A specific infant cannot have 8.4 IHEs in 1 h, but a group of infants can experience 8.4 IHEs per hour on average. This facilitates interpretation: if we observe 100 infants treated with usual care for 1 h each, we expect 100 infants × 1 h × 8.4 IHEs per infant‐hour = 840 IHEs.

## 4 HOW DO TRIALS ESTIMATE RATES?

Poisson regression can be used if events occur randomly at a constant rate independently of each other. This is often implausible: one event may change the rate of subsequent events in the same participant. This can lead to *overdispersion* (event counts varying more than the Poisson distribution allows). Alternatively, negative binomial regression, for example, addresses overdispersion. Counts are *zero‐inflated* if there is a subpopulation contributing an *excess* of zero counts due to low event risk. Such data can be analyzed using mixture distributions. Reviewers should evaluate the distributional assumptions supporting trial analyses and may consider downgrading for risk of bias if the assumptions are disputable [3].

## 5 HOW DO TRIALS QUANTIFY TREATMENT EFFECT FOR COUNT DATA?

*Rate differences* and *rate ratios* compare rates between arms and hence quantify treatment effect. Rate difference is the rate in one trial arm minus the rate in another trial arm. Rate ratio is the rate in one trial arm divided by the rate in another trial arm. No effect is represented by a difference of zero and a ratio of one.

Rhein compared caffeine discontinuation (control) to continuation (intervention). At 35 weeks postmenstrual age, Rhein estimated rates of 8.4 and 3.6 IHEs per infant‐hour (see Rhein's table 3), respectively, giving a rate difference of 3.6−8.4 = −4.8 IH events per infant‐hour, or a rate ratio of 3.6/8.4 = 0.43 (no units), favoring continuation.

For the moment, assume that IHE rate is constant over infants and time. The rate ratio 0.43 can be interpreted as follows. If a specific infant would experience 10 IHEs per hour treated with control, they would be expected to experience 0.43 × 10 IHEs = 4.3 IHEs per hour under the intervention. Because an infant cannot experience fractional IHEs, this could be reported as 4–5 IHEs per hour. Such calculations can be used to populate anticipated rate columns of summary of findings tables.

The rate and variance of a Poisson distribution are equal. The variances corresponding to the standard deviations reported by Rhein are larger than the rates (e.g., $4.3^{2}>3.6$; see Rhein's table 3), suggesting the constant rate assumption is invalid due to overdispersion. The interpretation above is therefore simplified, but hopefully useful for this tutorial.

We presented estimates without confidence intervals to aid understanding, but trials should report these [4], they are necessary for meta‐analysis, and should be reported in reviews.

## 6 SHOULD WE META‐ANALYZE RATE DIFFERENCES OR RATE RATIOS?

We recommend rate ratios. A rate difference has the same units of exposure as its rates. This can be a problem if trials do not use a common unit of exposure: meta‐analyzing quantities with different units will give a nonsensical result. While rate differences can be converted to a common scale (analogous to standardized mean difference), differences typically generalize less well than ratios. Rate ratios are unitless (the units of exposure cancel in the division), so can often be meta‐analyzed even if trials used different units of exposure. This tutorial focuses on rate ratios.

## 7 HOW DO WE META‐ANALYZE RESULTS FROM TRIALS THAT USE COUNT OUTCOMES?

Three broad approaches can be used for meta‐analysis: generic inverse‐variance (GIV), methods based on the Poisson distribution, and if individual participant data (IPD) are available, it can be meta‐analyzed. GIV meta‐analysis is preferred when trials report rate ratios, which can be meta‐analyzed in the same way as other ratios (Cochrane Handbook §10.3–§10.3.3). Poisson methods are used when total event counts and total exposures are available for each trial arm [5]. We focus on GIV meta‐analysis because it is supported by RevMan Web and other software.

## 8 HOW TO EXTRACT DATA FOR GENERIC INVERSE VARIANCE META‐ANALYSIS OF RATE RATIO?

If you are extracting numbers of events, and there are more events in a trial arm or subgroup than participants, the outcome cannot be dichotomous. Dichotomous events happen at most once in each participant. They may be counts. However, you cannot assume you are *not* dealing with counts if a trial arm or subgroup has fewer events than participants. Do not enter arm‐wise event counts and sample sizes as if the outcome is dichotomous.

Table 1 explains how trial results can be entered into a GIV meta‐analysis as log rate ratios (see supplementary materials for derivations). Prefer methods closer to the top of the table, which require fewer or weaker assumptions. A meta‐analysis result obtained on the log rate ratio scale will need to be exponentiated to express the result as a rate ratio. This may be done automatically by your meta‐analysis software, but if not, you may need to use an appropriate option or perform the calculation yourself.

**Table 1:** How to meta‐analyze trials that use count outcomes.

		Quantities to enter into GIV meta‐analysis	
	Trial reports	Log rate ratio	CI or SE for log rate ratio
1	•Rate ratio RR •Confidence interval (LtoU)	lnRR	CI=(logLtologU)
	**Example:** 0.43 (95% CI 0.42 to 0.44)	−0.84	95%CI=(−0.87to−0.81)
2	•Arm‐wise rates $r_{1}$ and $r_{2}$ •Arm‐wise total event counts $n_{1}$ and $n_{2}$	$\ln\frac{r_{1}}{r_{2}}$	$\text{SE}=\sqrt{\frac{1}{n_{1}}+\frac{1}{n_{2}}}$
	**Example:** $r_{1}=3.6$ IHEs/infant‐hour and $r_{2}=8.4$ IHEs/infant‐hour $n_{1}=7821$ IHEs and $n_{2}=14534$ IHEs	−0.85	SE=0.014
3	•Arm‐wise total event counts $n_{1}$ and $n_{2}$ •Arm‐wise total exposures $t_{1}$ and $t_{2}$	$\ln\left(\frac{n_{1}/t_{1}}{n_{2}/t_{2}}\right)$	$\text{SE}=\sqrt{\frac{1}{n_{1}}+\frac{1}{n_{2}}}$
	**Example:** $n_{1}=7821$ IHEs and $n_{2}=14534$ IHEs $t_{1}=2172.4$ hours and $t_{2}=1730.2$ hours	−0.85	SE=0.014
4	•Arm‐wise total event counts $n_{1}$ and $n_{2}$ •Arm‐wise mean exposures $e_{1}$ and $e_{2}$ •Arm‐wise sample sizes $N_{1}$ and $N_{2}$	$\ln\left(\frac{n_{1}/\left(e_{1}\times N_{1}\right)}{n_{2}/\left(e_{2}\times N_{2}\right)}\right)$	$\text{SE}=\sqrt{\frac{1}{n_{1}}+\frac{1}{n_{2}}}$
	**Example:** $n_{1}=7821$ IHEs and $n_{2}=14534$ IHEs $e_{1}=51.7$ hours and $e_{2}=32.6$ hours $N_{1}=42$ infants and $N_{2}=53$ infants	−0.85	SE=0.014
5	•Arm‐wise total event counts $n_{1}$ and $n_{2}$ •Arm‐wise sample sizes $N_{1}$ and $N_{2}$ •Hazard ratio HR and its standard error ${\text{SE}}_{\ln\text{HR}}$ (see notes)	$\ln\frac{n_{1}/N_{1}}{n_{2}/N_{2}}-\ln\text{HR}$	$\text{SE}=\sqrt{\frac{1}{n_{1}}+\frac{1}{n_{2}}+{\text{SE}}_{\ln\;\text{HR}}^{2}}$
	**Example:** $n_{1}=7821$ IHEs and $n_{2}=14534$ IHEs $N_{1}=42$ infants and $N_{2}=53$ infants HR=1.59(95%CI1.51to1.67)	−0.85	SE=0.030

*Note*: RR: rate ratio; CI: confidence interval; SE: standard error; L and U: lower and upper CI bounds; IHEs: intermittent hypoxia episodes. Note that SE depends on numbers of events, not sample sizes. For method 1, if your software requires a SE and does not allow you to enter a CI, use the method from §6.3.1 of the Cochrane Handbook to impute a SE. For method 5, the hazard ratio must be for an event coincident with end of exposure to event risk (e.g., death, study withdrawal) and must have the opposite direction of effect as the rate ratio, so may need to be inverted. If a CI is reported for HR, as in the example, the SE may be imputed per §6.3.1 of the Cochrane Handbook. Supplementary materials provide more details on the hazard ratio method, including its assumptions. The SE for the example for method 5 is larger (less precise) than for the other examples because it accounts for the uncertainty on the estimate of HR.

Reviewers often need to meta‐analyze AEs, but many trials do not report treatment effect estimates for safety. AEs are commonly reported as arm‐wise totals of AEs that may occur at most once (dichotomous outcomes) or multiple times (count outcomes).

Think carefully before entering AE or other event totals in a meta‐analysis if risk exposure differed between participants (e.g., events were assessed from randomization until death rather than over a predefined period): even if outcomes are dichotomous, it may be better to a estimate rate ratio. If there is no true difference in event risk or rate between intervention and control, but control participants die sooner and hence experience fewer events than intervention participants, a risk or odds ratio may incorrectly suggest control is safer than intervention (type I error). A type II error can occur if a true difference in risk or rate is obscured by differences in exposures that result in similar numbers of events in each arm. This problem is addressed by method 5 (see Table 1), which estimates risk ratio using a hazard ratio for a separate outcome coincident with the end of exposure to event risk. Its assumptions may not be valid or testable, so sensitivity analyses should be considered, and reviewers may consider downgrading based on their results [6]. Reviewers should read the supplementary materials before using the method.

## 9 COMMON MISUNDERSTANDINGS AND SUBTLETIES

Protocols that plan to meta‐analyze events that can occur multiple times in each participant, such as AEs, should plan to meta‐analyze counts to avoid the errors explained in Section 8.

Terminology can cause misunderstandings, such as using “rate” when “risk” is meant. Non‐statisticians may find it challenging to distinguish, should seek clarification, and avoid using these terms interchangeably.

The Cochrane Handbook advises on meta‐analyzing counts in §10.8, noting the constant rate assumption. However, counts can be analyzed without this assumption. It advises meta‐analyzing counts as continuous data. This is reasonable for large counts, which are approximately normally distributed, but the approximation is poor for small counts (e.g., rarer events, smaller trials).

## APPENDIX — COUNTING FRECKLES IN A TRIAL TO COMPARE SUNSCREEN USE TO NO SUNSCREEN

Figure 1 suggested that freckle count could be used as an outcome in a trial comparing sunscreen use to no sunscreen and asked you to consider the advantages and disadvantages of the outcome, and its use in meta‐analysis. We chose freckles so that we could present the concept of a count visually, but we would probably not suggest this outcome be used in a real trial. The main advantage of the outcome is that it is probably fast and easy to assess at low cost. The main disadvantages are that: some people do not develop freckles so the outcome would exclude certain populations; freckles grow and merge which may make outcome assessment difficult and introduce unnecessary variance; and counting freckles in a 10 cm² skin region leads to the question about which skin region should be sampled, and might allow trialists to manipulate results if they are unblinded and free to choose skin regions. If multiple trials used this outcome, but sampled different regions of skin, defined and counted freckles in different ways, and assessed the outcome at different follow‐up times, then substantial heterogeneity could result, possibly making the results of the meta‐analysis unclear. It may be better for trials on sunscreen to use patient‐important outcomes such as time‐to‐diagnosis (e.g., of melanoma), or using a tool like the Dermatology Life Quality Index [7].

## FURTHER READING AND ONLINE CONTENT

More information on counts and rates can be found in Chapter 6 of The Cochrane Handbook for Systematic Reviews of Interventions [2]. Cochrane Training has produced a micro‐learning module to accompany this article (https://links.cochrane.org/cesm/tutorials/count-outcomes). An Excel file is provided for performing the calculations presented in Table 1. Some mathematical details and advice for reviewers that use hazard ratios to impute risk ratios are presented in supplementary materials.

## AUTHOR CONTRIBUTIONS

**Christopher James Rose**: Conceptualization; Methodology; Project administration; Writing—original draft; Writing—review and editing. **Milena Geist**: Writing—review and editing. **Matteo Bruschettini**: Conceptualization; Writing—review and editing.

## CONFLICT OF INTEREST STATEMENT

The authors declare no conflicts of interest.

## PEER REVIEW

The peer review history for this article is available at https://www.webofscience.com/api/gateway/wos/peer-review/10.1002/cesm.70022.

## Supporting information

Supporting information.

Supporting information.

## Data Availability

Data sharing is not applicable to this article as no new data were created or analyzed in this study. The trial data used in our examples are available in reference [1].
