# Supplementary material for: Count data, rates, rate differences, and rate ratios in meta‐analysis: A tutorial
Source: Cochrane Evid Synth Methods. 2025 Feb 28;3(2):e70022. doi: 10.1002/cesm.70022 (PMC12463115; doi:10.1002/cesm.70022)
Supplement: Supplementary file 1 — Supporting information. [file CESM-3-e70022-s001.docx]

# Count data, rates, rate differences, and rate ratios in meta-analysis: Supplementary materials

## Introduction

Statistical methods are usually presented in introductory courses for non-statisticians as opaque formulas that, in many cases, can apparently be used in some situations, must not be used in others, and involve assumptions and approximations that may seem mysterious or untrustworthy. This document provides a little mathematical detail on some of the methods described in the tutorial. The aim is to allow readers with mathematical education at about the university entry level to understand more of the statistical thinking behind the methods presented in the tutorial. We will however not attempt explain all the ideas we rely on, so interested readers are directed to other teaching material. Mathematical statisticians will need to forgive us for any lack of rigor and abuse of notation. They will already understand everything we have to say and are not the target audience.

Section 1 explains how to estimate a log rate ratio from arm-wise total counts and total exposures, and in particular how sampling variances and SEs on log rate and log rate ratio are computed. This is essential for generic inverse-variance meta-analysis because study estimates are “weighted” by inverse sampling variance. Section 2 explains how a hazard ratio can be used to estimate a log rate ratio when total exposure is not reported. It is not necessary to read and understand section 1 before reading section 2.

## How do we estimate a rate ratio from total counts and total exposures?

A rate ratio is simply the ratio of two rates, one for each treatment. However, if we estimate anything using data, there is uncertainty due to sampling error (and perhaps other sources we will not consider). To perform a generic inverse-variance meta-analysis of rate ratios, it is necessary to calculate a log rate ratio and its standard error (SE) for each trial. We often report estimates as a point (a number) and a 95% confidence interval around that point. A confidence interval can be calculated from a standard error (SE). A SE is simply the expected distance between an estimate and the unknown true value being estimated. It is impossible to calculate a distance between two values if one is unknown, but as we’ll see it is possible to calculate the *expected value* of this distance.

Our strategy to estimate a ratio rate comparing two treatments is as follows:

1. Work out how to estimate an expected *count* for a single treatment (i.e., calculate a point estimate and its SE).
2. Work out how to transform a point estimate and its SE of an expected count into a a point estimate and its SE of an expected *log rate*.
3. Work out how to take two point estimates and their SEs of log rate (one for each of two treatments being compared), and compute the corresponding log rate ratio and its SE, which can be used in a meta-analysis.

### How do we estimate an expected count?

Estimation requires choosing an estimator (an estimation method). A common choice is maximum likelihood. A maximum likelihood estimator finds the value of a target parameter (expected count, in our case) that would be most likely to have generated the observed data. The likelihood of the observed data is modelled using a probability distribution, which is called a likelihood function in this context. The likelihood function is parameterized in terms of the target parameter. Conceptually, the likelihoods for all possible values of the parameter are evaluated using a set of observed data. The value of the parameter with maximum likelihood is chosen as the best estimate of the parameter. However, sampling error is likely to have caused the observed data to give a maximum likelihood estimate that is not perfectly equal to the true value we are trying to estimate. It is therefore also necessary to estimate how far our estimate is likely to be from its true value (i.e., to calculate its SE).

Assume that a total count of $n$ events occurred in one of the trial arms. We will consider the case where the events were generated by a Poisson distribution, which assumes that events occur randomly at a constant rate and that events occur independently of one another. The probability mass function for the Poisson distribution, and the likelihood function we want to maximize, is:

| $P\left( n;\theta\right)=\frac{\theta^{n}e^{-\theta}}{n!}$ | (1) |
| --- | --- |

where $\theta$ is mean count, the parameter we want to estimate from the total count, $n$, observed in the trial It is difficult to find the value of $\theta$ that maximizes the likelihood function given in equation (1) directly. However, the problem can be made easier by taking the logarithm of the likelihood function given in equation (1), and maximizing the resulting log likelihood function. Taking the logarithm does not change the value of $\theta$ that maximizes the likelihood function, it simply makes the problem easier to solve, as well as having some statistical and computational properties that are useful, but which we will not discuss further. Taking the logarithm of equation (1) gives the log likelihood function we want to maximize:

| $\mathcal{l}\left( \theta\right)=n\ln\theta-\theta-\ln n!$ | (2) |
| --- | --- |

A function can be maximized by finding where it is flat (i.e., has zero gradient, which means the function is not increasing or decreasing in value) and ensuring that this location corresponds to a maximum of the function rather than a minimum. This can be achieved by taking the first derivative of the function (i.e., finding an expression for its gradient). The first derivative of the log likelihood function is:

| $\frac{d}{d\theta}\mathcal{l}\left( \theta\right)=\frac{n}{\theta}-1$ | (3) |
| --- | --- |

The value of $\theta$ at which $\mathcal{l}\left( \theta\right)$ is flat can be found by setting equation (3) to zero and then solving for $\theta$:

| $\frac{n}{\theta}-1=0\Longrightarrow\theta=n$ | (4) |
| --- | --- |

In other words, the maximum likelihood estimate of the expected count, $\theta$, is simply the observed count, $n$. We verify that the solution is a maximum rather than a minimum by checking that the second derivative of the log likelihood function is negative at the solution:

| $\frac{d^{2}}{d\theta^{2}}\mathcal{l}\left( \theta=n \right)=-\frac{1}{n}<0$ | (5) |
| --- | --- |

We now need to calculate the SE for the maximum likelihood estimate of $\theta$. A SE on $\theta$ is the square root of the sampling variance of $\theta$, $\text{Var}\left[ \theta\right]$:

| $\text{SE}_{\theta}=\sqrt{\text{Var}\left[ \theta\right]}$ | (6) |
| --- | --- |

Sampling variance is the reciprocal of something called Fisher information, $I(\theta)$:

| $\text{Var}\left[ \theta\right]=\frac{1}{I(\theta)}$ | (7) |
| --- | --- |

To avoid a detailed mathematical aside, we will simply point out that Fisher information can be computed via the second derivative of the log likelihood function as follows:

| $I\left( \theta\right)=-E\left[ \left. \frac{d^{2}}{d\theta^{2}}\mathcal{l}\left( \theta\right) \right\vert\theta\right]=\frac{1}{\theta}$ | (8) |
| --- | --- |

Sampling variance on $\theta$ at the maximum likelihood solution $\theta=n$ is therefore $n$, so $\text{SE}_{\theta}=\sqrt{n}$. However, recall that so far, we have only estimated expected count, not expected log rate.

### How do we estimate an expected log rate?

We can estimate an expected rate as the expected count divided by total exposure. Note that we have assumed that total exposure is known (e.g., reported by a trial), so there is no statistical uncertainty on this quantity. The maximum likelihood estimate of expected rate is simply:

| $r=\frac{\theta}{t}$ | (9) |
| --- | --- |

where $t$ is total exposure, and $\theta$ is expected count, as above. However, to perform a meta-analysis, we need to compute log rates and their SEs. To make the following mathematics easier, we will use a function $f$ that computes a log rate for an expected count, $\theta$, and total exposure, $t$:

| $f\left( \theta,t \right)=\ln\frac{\theta}{t}=\ln\theta-\ln t$ | (10) |
| --- | --- |

With $\theta$ estimated by $n$, the maximum likelihood estimate of log rate is $f(n,t)$. However, we also need to compute SE on this log rate. An approximate SE can be computed by applying the delta method. The delta method approximates the variance of a function of an estimated parameter using a first order Taylor series:

| $\text{Var}\left[ f(\theta,t) \right]\approx\text{Var}\left[ \theta\right]\left( \frac{\partial}{\partial\theta}f(\theta,t) \right)^{2}$ | (11) |
| --- | --- |

We know $\text{Var}\left[ \theta\right]=n$, and $\frac{\partial}{\partial\theta}f\left( \theta,t \right)=\frac{1}{\theta}$, so the sampling variance on the maximum likelihood estimate of log rate, $\text{Var}\left[ f(\theta,t) \right]$, with $\theta$ estimated by $n$, is:

| $\text{Var}\left[ f(\theta,t) \right]=n\left( \frac{1}{n} \right)^{2}=\frac{1}{n}$ | (12) |
| --- | --- |

SE on log rate is therefore$\sqrt{\frac{1}{n}}$.

We can now obtain a maximum likelihood estimate of log rate and the corresponding SE, and we now want to compute a log risk ratio comparing two treatments and the SE if that log risk ratio.

### How do we estimate a log rate ratio comparing two treatments?

Plugging the maximum likelihood estimate of a count, $\theta=n$, into equation (10) gives the maximum likelihood estimate of log rate, $\ln r=\ln n-\ln t$, which has sampling variance $\frac{1}{n}$. Assume a trial reported counts of $n_{1}$for treatment 1 and $n_{2}$for treatment 2, and total exposures $t_{1}$ and $t_{2}$. The maximum likelihood estimates of log rate are therefore $\ln r_{1}=\ln n_{1}-\ln t_{1}$ and $\ln r_{2}=\ln n_{2}-\ln t_{2}$ for treatments 1 and 2, respectively. The log rate ratio is therefore:

| $\ln\frac{r_{1}}{r_{2}}=\ln r_{1}-\ln r_{2}=\left( \ln n_{1}-\ln t_{1} \right)-\left( \ln n_{2}-\ln t_{2} \right)$ | (13) |
| --- | --- |

The sampling variances on the log rates are $\frac{1}{n_{1}}$ and $\frac{1}{n_{2}}$. Note from equation (13) that log rate ratio is a difference on the log scale. The sampling variance for a difference is the sum of the respective variances:

| $\text{Var}\left[ \ln r_{1}-\ln r_{2} \right]=\frac{1}{n_{1}}+\frac{1}{n_{2}}$ | (14) |
| --- | --- |

SE on log rate ratio is therefore $\sqrt{\frac{1}{n_{1}}+\frac{1}{n_{2}}}$, as given in the tutorial.

If no events occur in one or both trial arms, then the SE on log rate ratio is infinitely large, which is somewhat of an overestimate and would give the study zero “weight” in a generic inverse-variance meta-analysis (as $\frac{1}{\infty}=0$). This problem is due to the use of the delta method in equation (11), which only provides an approximation of sampling variance. Alternative methods are available to address this issue.

This concludes the explanation of how log risk ratio and its SE can be computed given total counts and total exposures.

## Using a hazard ratio to impute rate ratio when total exposure is not reported

Section 8 of the tutorial describes two scenarios where it may be preferable to estimate a rate ratio because risk exposure differed between participants (e.g., because events were assessed from randomization until an endpoint such as death that would be different for each participant):

1. A trial reports arm-wise totals of events that could occur zero or once for each participant (dichotomous outcomes).
2. A trial reports arm-wise totals of events that could occur zero, once, or multiple times in each participant (count outcomes).

It is inappropriate to estimate an odds ratio, risk ratio, or risk difference for scenario 2 because the outcomes are not dichotomous, they are counts.

It is inappropriate to quantify treatment effect for scenario 1 using an odds ratio, risk ratio, or risk difference if events could happen at any time between randomization and some endpoint that can vary by participant. This is true for two reasons: first, a risk must be defined in terms of an exposure (e.g., time period) that is common to all participants; second events cannot occur in participants after they reach the endpoint.

Section 8 of the tutorial describes how type I and II errors — i.e., biases — can occur in scenario 1. As a concrete example, imagine a trial that compared two medicines that are each taken by swallowing a pill, in which participants who take the intervention survive longer than participants who take the control. Further, imagine that the two types of pill are identically sized and shaped, and that the event of interest is choking on a pill one or more times (a dichotomous outcome). It would be reasonable to expect the risk of choking to be approximately the same each time a pill is taken, and that there would be no difference in safety between two outwardly identical pills. Let us now assume that that is indeed true. Because intervention participants live longer than control participants, they would have more opportunities to choke on a pill (i.e., greater exposure). The trial is likely to report more events for intervention patients. If an odds ratio, risk ratio, or risk difference is used to quantify treatment effect for this safety outcome, then the intervention would appear to be less safe, even though the risk of choking by taking a single pill is identical for the two treatments. This issue can be considered as a form of immortal time bias (only survivors can experience events) and from the perspective of intercurrent events (see the ICH E9(R1) addendum on estimands in clinical trials). It may be more appropriate to estimate a rate ratio in this scenario, in which events are counted on groups of people rather than individuals in a way that accounts for differences in exposure. Such a rate ratio would have a “health system” rather than an individual participants interpretation. Any decision to adopt the intervention versus the control would of course need to consider the trade-off between the costs of the additional events expected for the intervention with the benefit of longer survival.

Conversely, it may be appropriate to quantify treatment effect for scenario 1 using an odds ratio, risk ratio, or risk difference if events could only occur during a common period of exposure that begins at or very soon after randomization and during which it is reasonable to assume that the vast majority of participants would be exposed to event risk (e.g., still be alive). The exposure must begin at or very soon after randomization to avoid the immortal time bias mentioned above. The period of exposure must be common to all participants so that events and risks have the same interpretation across participants and trial arms, respectively. For example, imagine a trial comparing two different types of surgery in which the event of interest is 30-day postoperative infection. Provided no or very few participants died within 30 days of surgery, all participants would be exposed for the same time and the events could be meta-analyzed as dichotomous to estimate an odds ratio, risk ratio, or risk difference. The surgery and pill examples illustrate that it is important to clearly define the research question that the treatment effect estimate sought in a meta-analysis should answer (i.e., the estimand).

Section 8 of the tutorial presents method 5, which can be used to address the scenario in which exposures differ between participants. The method relies on an estimate of hazard ratio (HR) for an outcome that coincides with end of exposure (e.g., overall survival), which can be used with reported counts and sample sizes to impute a log risk ratio that accounts for the difference in total exposures. This imputation assumes that time to end of exposure is approximately exponentially distributed in the two trial arms. If this assumption is valid in one of the arms, and the proportional hazards assumption holds (i.e., the estimate of HR is trustworthy), then time to end of exposure will also be approximately exponentially distributed in the other arm. Log rate ratio can be imputed as:

| $\ln\frac{r_{1}}{r_{2}}\approx\ln\frac{{n_{1}}/{N_{1}}}{{n_{2}}/{N_{2}}}-\ln\text{HR}$ | (15) |
| --- | --- |

where $n_{1}$ and $N_{1}$ are the total counts and total exposures in for treatment 1, $n_{2}$ and $N_{2}$ are the total counts and total exposures in for treatment 2, and HR is the hazard ratio for an outcome that coincides with end of exposure. As we explain below, the HR must have the opposite direction of effect as the rate ratio (i.e., the numerator and denominator treatments are reversed in the HR).

Recall from method 3 of the tutorial that if a trial reports event counts $n_{1}$ and $n_{2}$, and total exposures $t_{1}$ and $t_{2}$, log rate ratio can be computed as $\ln\frac{r_{1}}{r_{2}}=ln \left( \frac{{n_{1}}/{t_{1}}}{{n_{2}}/{t_{2}}} \right)$, and that in method 4 the total exposures are imputed as $t_{1}=e_{1}\times N_{1}$ and $t_{2}=e_{2}\times N_{2}$, where $e_{1}$ and $e_{2}$ are average exposures for participants in the two arms. The problem we need to address is that $e_{1}$ and $e_{2}$ are not reported. We can rewrite the expression for log rate ratio as:

| $\ln\frac{r_{1}}{r_{2}}=\ln\left( \frac{{n_{1}}/{t_{1}}}{{n_{2}}/{t_{2}}} \right)=\ln\frac{{n_{1}}/\left( e_{1}\times N_{1} \right)}{{n_{2}}/\left( e_{2}\times N_{2} \right)}=\ln\frac{{n_{1}}/{N_{1}}}{{n_{2}}/{N_{2}}}-\ln\frac{e_{1}}{e_{2}}$ | (16) |
| --- | --- |

Notice that $e_{1}$ and $e_{2}$ are now separated from the known quantities on the right-hand side.

An exponential distribution with mean time to event $e_{1}$ has hazard function $h_{1}\left( t \right)=\frac{1}{e_{1}}$ where $t$ is a time variable, and similarly for an exponential with mean $e_{2}$. The hazard functions for two exponentials are proportional, which means they can be compared using a HR. The HR comparing hazard functions for treatments 1 and 2 is therefore:

| $\text{HR}=\frac{h_{2}\left( t \right)}{h_{1}\left( t \right)}=\frac{\frac{1}{e_{2}}}{\frac{1}{e_{1}}}=\frac{e_{1}}{e_{2}}$ | (17) |
| --- | --- |

If the assumptions above are valid, this HR can therefore be used in place of $\frac{e_{1}}{e_{2}}$ in equation (16), and it is not necessary to know the two expected exposures. This allows log risk ratio to be imputed in a way that accounts for differences in total exposures between trial arms.

Note that the HR used in equation (17) has the opposite direction to the risk ratio we are imputing (i.e., the HR has the hazard function for treatment 2 in its numerator, while the risk ratio has event rate for treatment 1 in its numerator). It is necessary to verify how published HRs are defined and to invert them if necessary.

The SE on log rate ratio needs to account for the uncertainty on the HR estimate as well as that arising from the counts. Recall from section 1 that the sampling variance for a difference is the sum of the respective variances. The SE on log rate ratio is therefore approximately:

| $\sqrt{\frac{1}{n_{1}}+\frac{1}{n_{2}}+\text{SE}_{\text{ln HR}}^{2}}$ | (18) |
| --- | --- |

where $\text{SE}_{\text{ln HR}}^{2}$ is the square of the standard error on log HR.

It is important to note that the exponential distribution assumption will not always be valid and that the proportional hazards assumption is often violated. The approach outlined above will therefore be subject to imputation bias, which could be large. It is therefore sensible to perform sensitivity analyses, for example to determine the degree to which including versus excluding trials for which this method is used, and the degree to which varying the estimate of HR and its SE, affect the overall meta-analytical estimate. Reviewers should prespecify such sensitivity analyses in protocols that aim to study count outcomes, such as adverse events, and depending on the results of the sensitivity analyses, may consider downgrading for bias.
